# Advancements in Heart Transplantation: Donor-Derived Cell-Free DNA as Next-Generation Biomarker

**DOI:** 10.7759/cureus.54018

**Published:** 2024-02-11

**Authors:** Pawel Borkowski, Nikita Singh, Natalia Borkowska

**Affiliations:** 1 Internal Medicine, Albert Einstein College of Medicine, Jacobi Medical Center, New York, USA; 2 Pediatrics, SPZOZ (Samodzielny Publiczny Zakład Opieki Zdrowotnej) Krotoszyn, Krotoszyn, POL

**Keywords:** heart failure, heart transplant, cardiology, dd-cfdna, cell free dna

## Abstract

Heart failure, particularly in its advanced stages, significantly impacts quality of life. Despite progress in Guideline-Directed Medical Therapy (GDMT) and invasive treatments, heart transplantation (HT) remains the primary option for severe cases. However, complications such as graft rejection present significant challenges that necessitate effective monitoring. Endomyocardial biopsy (EMB) is the gold standard for detecting rejection, but its invasive nature, associated risks, and healthcare costs have shifted interest in non-invasive techniques. Donor-derived cell-free DNA (dd-cfDNA) has gained attention as a promising non-invasive biomarker for monitoring graft rejection. Compared to EMB, dd-cfDNA detects graft rejection early and enables clinicians to adjust immunosuppression promptly. Despite its advantages, dd-cfDNA testing faces challenges, such as the need for specialized technology and potential inaccuracies due to other clinical conditions. Additionally, dd-cfDNA cannot yet differentiate between types of graft rejection, and its effectiveness in chronic rejection remains unclear. Research is ongoing to set precise standards for dd-cfDNA levels, which would enhance its diagnostic accuracy and help in clinical decisions. The article also points to the future of HT monitoring, which may involve combining dd-cfDNA with other biomarkers and integrating artificial intelligence to improve diagnostic capabilities and personalize patient care. Furthermore, it emphasizes both global and racial inequalities in dd-cfDNA testing and the ethical issues related to its use in transplant medicine.

## Introduction and background

Heart failure is defined as “a clinical syndrome with symptoms and/or signs caused by a structural and/or functional cardiac abnormality and corroborated by elevated natriuretic peptide levels and or objective evidence of pulmonary or systemic congestion” [1]. Advanced heart failure often results in severe symptoms and frequent hospitalizations, reducing patients' quality of life [2]. The management of heart failure emphasizes traditional clinical findings and incorporates patients' self-assessments. From the patient's perspective, factors related to the quality of life, e.g., exercise tolerance, mental well-being, and the rate of hospital readmissions, hold the utmost importance [2]. Patients with advanced heart failure may be appropriate candidates for advanced heart failure therapies, e.g., left ventricular assist device (LVAD), palliative care, palliative inotropic agents, and heart transplant (HT), which improve the quality of life [3]. Although there have been advancements in pharmacotherapy and mechanical circulatory support, HT (a surgical procedure that replaces a failing or diseased heart with a heart from a deceased donor) continues to be the treatment of choice for advanced heart failure [4].

The incidence of HT in the United States is notably increasing. For example, in 2021, 3,901 hearts were recovered for transplantation, which is a significant rise from the 3,597 cardiac transplants in 2019 [5,6]. HT effectively enhances survival and quality of life in patients with advanced heart failure [7,8]. However, it is associated with various complications that can adversely affect the outcomes. They are categorized into non-allograft-related complications (e.g. bleeding, infection, malignancy) and allograft-related complications, notably cardiac allograft rejection [9]. Graft rejection is classified by its timing (hyperacute, acute, chronic) and mechanism (cell-mediated versus antibody-mediated) [10]. Effective monitoring and early intervention are crucial for transplant recipients' long-term survival. Introduced in the early 1970s by Dr. Phillip Caves and Dr. Margaret Billingham, endomyocardial biopsy (EMB) - the removal of a small heart muscle tissue sample for examination - continues to be the gold standard for diagnosing heart transplant rejection [4,11]. While generally considered a low-risk procedure, studies by Fowles et al. and Saraiva et al. showed complication rates of less than 1% (over 4000 biopsies performed) and 0.71% (over 2000 biopsies performed), respectively [12,13]. Nevertheless, the invasive nature of EMB and its associated risks, such as arrhythmias, cardiac perforation, heart valve damage, vasovagal reactions, puncture site hematoma, or nerve injury, alongside potential sampling error and interobserver variability, cannot be overlooked [12,13]. As a result, there is an increasing interest in non-invasive methods for monitoring graft rejection. These methods not only facilitate early detection but also improve the quality of life for patients by minimizing the need for invasive procedures. The most promising non-invasive techniques are cardiac magnetic resonance imaging and circulating donor-derived cell-free DNA (dd-cfDNA) analysis [14]. The primary aim of this narrative review is to increase awareness of dd-cfDNA as an essential aspect of post-transplant monitoring.

## Review

Discovery and applications of circulating free DNA in medicine

The discovery of circulating free DNA (cfDNA), also known as cell-free DNA, by Mandel and Metais in 1948 marked an important milestone in medical science [15]. Since then, cfDNA has gained widespread attention for its potential as a noninvasive biomarker in medical research and diagnostics. cfDNA refers to extracellular DNA fragments released into body fluids during cellular destruction, such as necrosis or apoptosis [15,16]. The presence of cfDNA is observed in physiological conditions (e.g. pregnancy and immune system regulation) and pathological states (e.g., autoimmune diseases, cancer, and graft rejection) [16-20]. One of the most promising applications of cfDNA is in transplantology, specifically dd-cfDNA [21]. This form of cfDNA, originating from a transplanted organ, can be found in the recipient's plasma. When the immune system recognizes a transplanted organ as a foreign material, the recipient's immune response induces cellular damage in the donor organ, consequently elevating the levels of dd-cfDNA in the recipient's plasma. Sophisticated assays, such as next-generation sequencing or digital PCR, have been developed to differentiate dd-cfDNA from the genetic material originating from the organ donor and the transplant recipient and allow for the precise quantification of dd-cfDNA [22,23]. Therefore, advanced genomic techniques are highly effective for monitoring changes in the graft's condition.

Advantages of dd-cfDNA testing in enhancing transplant patient care

Like EMB, checking dd-cfDNA levels in transplant patients' blood is vital for detecting graft damage or rejection. Importantly, dd-cfDNA provides an earlier detection of graft rejection compared to traditional biopsy. In a study involving 171 participants, Agbor-Enoh et al. found that dd-cfDNA levels rise earlier than biopsy-confirmed diagnoses [20]. Specifically, they increase about 0.5 months before acute cellular rejection (ACR) and 3.2 months before antibody-mediated rejection (AMR) can be detected by biopsy. This enables clinicians to adjust immunosuppressive therapy promptly and extend graft life. This proactive approach has the potential to not only enhance patient outcomes but also contribute to optimizing healthcare resources. Additionally, dd-cfDNA monitoring could facilitate longitudinal monitoring of disease progression, allowing for more frequent and less invasive sample collection than biopsies. Consequently, the data could be tracked over time, offering a clear picture of graft status. The economic aspects of dd-cfDNA testing are also noteworthy. In 2019, the average cost for an outpatient EMB was around $7,918 [24]. In contrast, commercially available dd-cfDNA tests are priced at approximately $3,000 [25]. Furthermore, performing an EMB requires specialized medical skills, making it a more complex procedure [26]. Conversely, dd-cfDNA tests are less demanding regarding resources. Additionally, from a patient compliance perspective, dd-cfDNA testing offers significant benefits. This test's non-invasive nature reduces patient discomfort and provides reassurance through regular, reliable monitoring of graft health. This aspect of dd-cfDNA testing may improve adherence to monitoring protocols among patients. It also has the potential to enhance the patient-doctor relationship. As patients become more engaged in monitoring their health, they can actively participate in treatment plans, fostering a sense of empowerment and responsibility for their health. Finally, using dd-cfDNA in clinical settings could allow healthcare providers to customize immunosuppressive therapy for each patient, departing from a uniform treatment strategy [27].

Challenges and limitations of dd-cfDNA testing in heart transplantation

While dd-cfDNA testing in heart transplantation presents promising advantages, it also faces several limitations and challenges. First, the accuracy of dd-cfDNA testing varies based on the technology and methods employed [28]. Second, clinical scenarios that cause organ injury and DNA release, such as pulmonary embolism, infection, autoimmune conditions, ischemia, and concomitant cancer, may lead to elevated dd-cfDNA levels in the plasma [29,30]. Clinicians must consider these factors to ensure accurate dd-cfDNA interpretation. To mitigate some of these limitations, absolute dd-cfDNA quantification methods are recommended for more precise and reliable results [27]. Third, dd-cfDNA effectively detects ACR and AMR, yet it cannot distinguish between them. Supporting this, Huang et al.'s study revealed that dd-cfDNA levels were similar in patients with isolated AMR and those with combined CMR/AMR, indicating no significant distinction between the types [31]. Lastly, while dd-cfDNA helps detect acute rejection, its effectiveness in predicting or diagnosing chronic rejection must be clarified. Despite these challenges, continued research and technological advancements are expected to address these limitations, thus improving dd-cfDNA's effectiveness in heart transplant patient management. Additionally, including diverse populations in research is crucial to ensure the efficacy and accuracy of dd-cfDNA testing across different genetic backgrounds.

Future directions in enhancing dd-cfDNA diagnostic accuracy and application

Ongoing research is aimed at determining the ideal threshold for dd-cfDNA to improve its diagnostic accuracy. For instance, Agbor-Enoh et al. discovered that a threshold of 0.25% results in a 99% negative predictive value (NPV) for acute rejection, which could potentially reduce endomyocardial biopsies by 81% [20]. Similarly, Khush et al. identified a 0.2% cutoff correlating with a 97.1% NPV [32]. It is crucial to establish standardized thresholds to minimize variability in clinical decision-making. This standardization could ensure that healthcare professionals can consistently interpret test results, leading to more uniform and effective patient care. In addition, dd-cfDNA presents unique patterns that may indicate different types of graft rejection like ACR and AMR [33]. The treatment for ACR includes glucocorticoids, anti-thymocyte globulin (ATG), and adjustments in the immunosuppression regimen [34]. In contrast, the treatment for AMR typically includes glucocorticoids, plasmapheresis, intravenous immunoglobulin (IVIG), changes in the immunosuppression regimen, ATG, and bortezomib [35]. Therefore, differentiating between ACR and AMR is essential, as their treatment strategies differ [20]. Additionally, investigating genetic variations that affect dd-cfDNA levels could enable more personalized and precise threshold determinations. This strategy would tailor dd-cfDNA test interpretation to each patient's genetic profile, leading to more customized threshold settings. Furthermore, integrating dd-cfDNA analysis with established (e.g., troponins) or emerging (e.g., circulating micro-RNA) biomarkers could offer a more comprehensive assessment of graft health [36]. This holistic approach could enhance diagnostic accuracy and prognostic capabilities. In addition, applying artificial intelligence to dd-cfDNA data could yield powerful predictive models for graft survival and rejection. These models could guide clinical decision-making and identify at-risk patients sooner. Finally, making dd-cfDNA testing more cost-effective and accessible could lead to its broader use in clinical practice, benefiting a larger group of transplant recipients.

Global accessibility and disparities in healthcare

The global accessibility of cfDNA testing exhibits significant disparities [37]. Wealthy nations are likely to adopt this advanced technology quickly. In contrast, developing countries face barriers like the lack of specialized equipment and limited technical expertise, leading to gaps in health outcomes across populations. Addressing these disparities could involve creating cost-effective dd-cfDNA tests, international technology transfer, and implementing training programs to improve local capabilities. In addition, global disparities in insurance coverage for advanced testing like dd-cfDNA could exacerbate healthcare inequalities.

The Institute of Medicine's seminal report revealed that racial and ethnic minorities often receive lower-quality health care than white Americans, regardless of similar income and insurance coverage [38]. This disparity extends beyond general healthcare and is particularly pronounced in specialized areas such as heart transplantation. For instance, a comprehensive study by Liu et al., encompassing 39,075 adult primary heart transplant recipients from 1987 to 2009, revealed a significant difference in five-year mortality rates between African-American and Caucasian recipients (35.7% versus 26.5%, respectively) [39]. This suggests systemic factors affecting post-transplant outcomes based on race. Further supporting this, Doshi et al. found a higher proportion of AMR in African-American patients compared to non-African-American patients (21% versus 9%) [40]. In cancer care, for example, racial disparities are well-documented in the context of genetic testing, where African-American individuals are less likely to utilize these services compared to their Caucasian counterparts [41]. This trend suggests a broader pattern of disparity for genetic testing that could extend to other areas, including heart transplantation monitoring with dd-cfDNA.

Ethical considerations

The integration of dd-cfDNA testing in transplant medicine brings complex ethical considerations. The use of dd-cfDNA raises concerns about genetic privacy and the potential misuse of sensitive genetic information. While there are existing legal frameworks, such as the Health Insurance Portability and Accountability Act (HIPAA) and the Genetic Information Nondiscrimination Act (GINA), which provide some level of protection and guidelines, their application mainly focuses on areas like paternity testing, prenatal testing, genetic disorders, oncology, and criminology [42]. In these fields, well-established protocols and laws regulate the use and disclosure of genetic information. Given that dd-cfDNA involves analyzing DNA fragments from a transplanted organ, it raises privacy concerns that current regulations may not entirely address. These concerns include the risk of revealing genetic information not only about the recipient but about the donor as well. Patients and healthcare providers must also be aware of these ethical considerations as dd-cfDNA testing becomes more common.

## Conclusions

In conclusion, integrating dd-cfDNA analysis in heart transplantation is a significant step in patient care and management. This non-invasive biomarker offers several advantages, including early detection of graft rejection, reduced need for invasive procedures, dynamic way to monitor graft health and response to treatment, and potential for cost savings. Its real-time monitoring of graft health revolutionizes post-transplant care, improving patient comfort and follow-up adherence. However, dd-cfDNA testing faces challenges such as privacy concerns, the need for specialized technology, and the potential for false positives due to other clinical conditions. The variability in cutoff thresholds and the inability to distinguish between different types of rejection also highlight the need for standardization and continued research. Future directions include integrating dd-cfDNA with other biomarkers and applying artificial intelligence to assess graft health. As research advances, dd-cfDNA testing will likely become more affordable and widely accessible, significantly enhancing its use in clinical practice and transplant medicine. In addition, this article highlights the global and racial disparities in dd-cfDNA testing and associated ethical concerns in transplant medicine.
